# Association between hepatitis C virus and oral lichen planus: HCV and oral Lichen Planus

**Published:** 2011-02-01

**Authors:** Mônica Ghislaine Oliveira Alves, Janete Dias Almeida, Luiz Antonio Guimarães Cabral

**Affiliations:** 1 University of Estadual Paulista, São José dos Campos Dental School, Department of Biosciences and Oral Diagnosis, São José dos Campos, São Paulo, Brazil

**Keywords:** Lichen planus, Hepatitis C virus

Dear Editor,

We read with great interest the article, "Hepatitis C virus and Lichen Planus: The real association" by Mahboobi et al. [1] because our practice serves a large group of patients with Oral Lichen Planus (OLP). OLP has been associated with chronic liver diseases, particularly those that have Hepatitis C Virus (HCV) [2] as a possible etiology. The association between HCV and Lichen Planus has been described in the literature, especially among patients of Mediterranean origin, but is not generally observed in patients from Northern Europe, indicating a strong geographic relationship [2]. Carrozzo et al. [2] reported that HCV-associated Lichen Planus appears to be a distinct subset among Lichen Planus conditions and is particularly associated with the HLA class II allele, HLA-DR6. This fact may explain in part the peculiar geographic heterogeneity seen in HCV-associated Lichen Planus. Given that HCV infection can be asymptomatic, screening patients with Lichen Planus for this virus is important because it permits an early diagnosis and a better prognosis. Therefore, we request serological tests for patients diagnosed with OLP and encourage this practice. At our practice, none of the patients diagnosed with OLP have been HCV positive [3]. However, the number of patients in our study is not sufficient to demonstrate a positive association, which might be explained by the ethnic diversity of Brazil, although a geographic or ethnic correlation is difficult to establish. The pathogenesis of OLP induced by HCV is uncertain, but two hypotheses have been raised to explain the mechanism of the triggering of OLP by HCV. The first hypothesis suggests that virus replication is associated with the oral epithelium and thus contributes directly to the development of lesions. The second hypothesis proposes that the high mutation rate of the virus results in repeated activation of immune cells, increasing the probability of crossreaction with its own tissue and, consequently, the risk of autoimmune disease. In certain genotypes, crossreactivity that activates immune cells against epithelial cells is favored [4]. According to Arrieta et al. [5], HCV infection is not a direct causal factor of OLP because replication of HCV was observed in both mucosa with and without OLP. In addition, the authors found a mononuclear cell infiltrate around the epithelial cells of HCV-seropositive patients with and without OLP. However, the authors did not rule out the possibility of HCV inducing changes in the host that may have led to an autoimmune response. Michele et al. [6] found no clear association between OLP and chronic hepatitis C. These authors postulated that this possible association mainly depends on the frequency of each disease in the population, which would explain the wide geographic variation. However, Del Olmo et al. [7] concluded that HCV plays a role in the etiopathogenesis of chronic liver diseases documented in patients with OLP and that treatment of the disease with IFN-α, which inhibits virus replication, may lead to the development of a lichenoid reaction to this drug. Despite the controversy in the literature regarding the association between OLP and hepatitis caused by HCV, we partly agree with Mahboobi et al. [1] that screening patients with OLP is of marked importance for the diagnosis of HCV infection given that the latter is usually indolent and can cause serious complications in patients if left untreated.
